# Supplementary material for: Effectiveness of Pilates and Yoga to improve bone density in adult women: A systematic review and meta-analysis
Source: PLoS One. 2021 May 7;16(5):e0251391. doi: 10.1371/journal.pone.0251391 (PMC8104420; doi:10.1371/journal.pone.0251391)
Supplement: S1 Fig — Green circles: low risk of bias; yellow circles: some concerns; red circles: high risk of bias. (PDF) [file pone.0251391.s001.pdf]

	Randomisation process	Deviations from the intended interventions	Missing outcomes	Measurement of the outcome	Selection of reported results	Overall bias
Irez et al, 2009 [34]	?	+	+	+	+	?
Bezerra et al, 2010 [30]	?	+	+	+	+	?
Angin et al, 2015 [17]	?	+	+	+	+	?
Kim et al, 2015 [35]	-	+	-	?	?	-
Oliveira et al, 2018 [18]	+	+	+	-	+	-

S1 Fig. Quality Assessment for RCTs (RoB 2.0).

+ : Low risk of bias; ? : Some concerns; - : High risk of bias
