# Supplementary material for: Effectiveness of Pilates and Yoga to improve bone density in adult women: A systematic review and meta-analysis
Source: PLoS One. 2021 May 7;16(5):e0251391. doi: 10.1371/journal.pone.0251391 (PMC8104420; doi:10.1371/journal.pone.0251391)
Supplement: S2 Fig — Green circles: strong score; yellow circles: moderate score; red circles: weak score. (PDF) [file pone.0251391.s002.pdf]

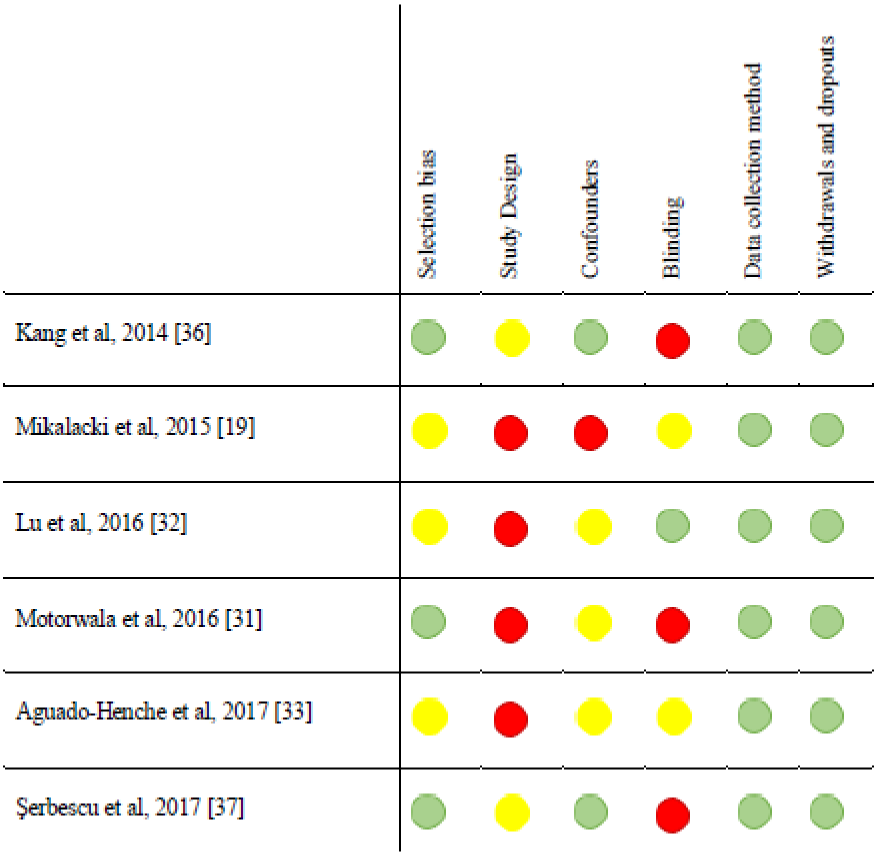

S2 Fig. Quality Assessment for non-RCTs and pre-post studies.

: Strong; : Weak; : Moderate.
