# Supplementary figures and images for: Effectiveness of Pilates and Yoga to improve bone density in adult women: A systematic review and meta-analysis
Source: PLoS One. 2021 May 7;16(5):e0251391. doi: 10.1371/journal.pone.0251391 (PMC8104420; doi:10.1371/journal.pone.0251391)

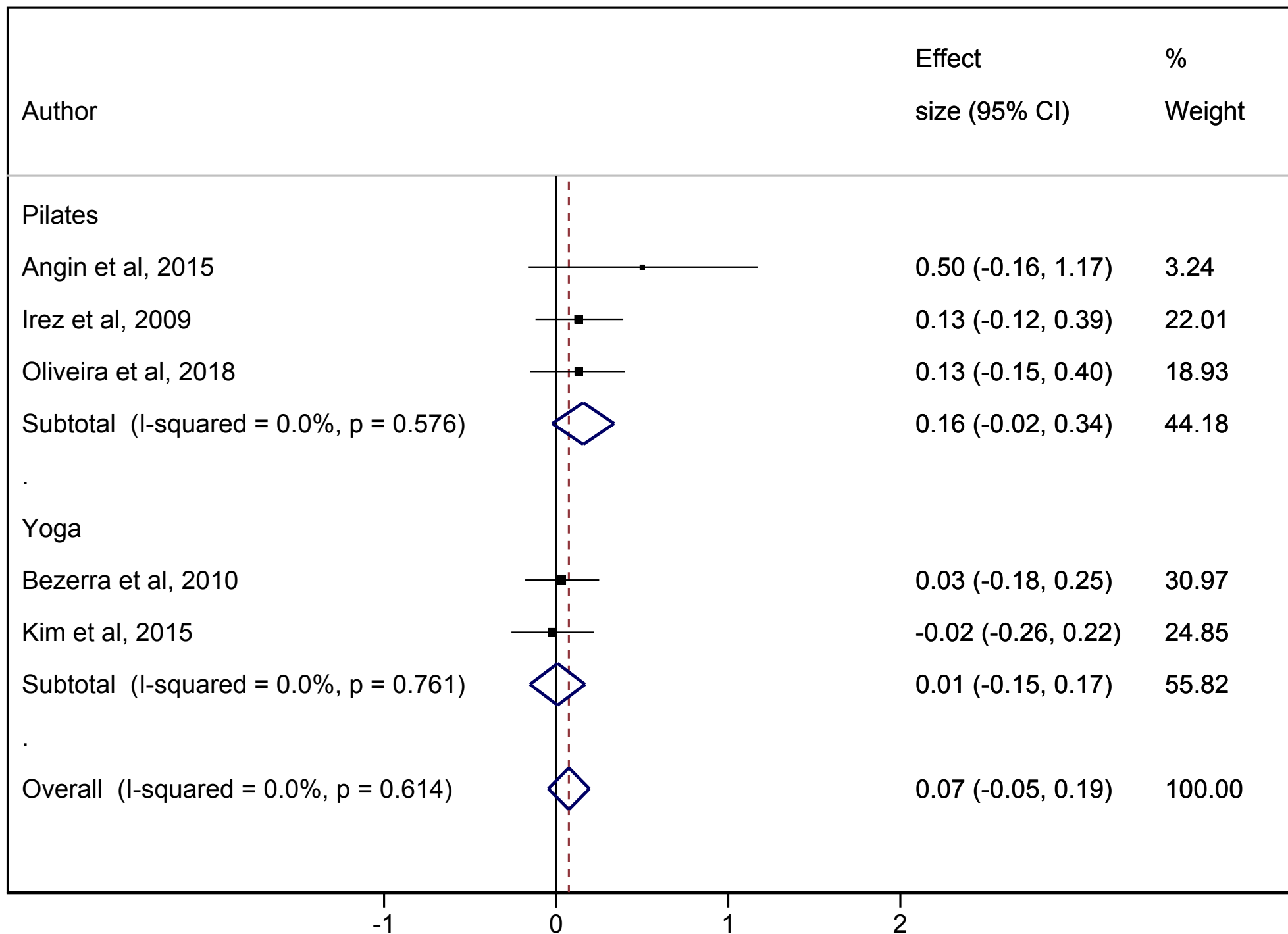

Supplement: S3 Fig — (PDF) [file pone.0251391.s003.pdf]

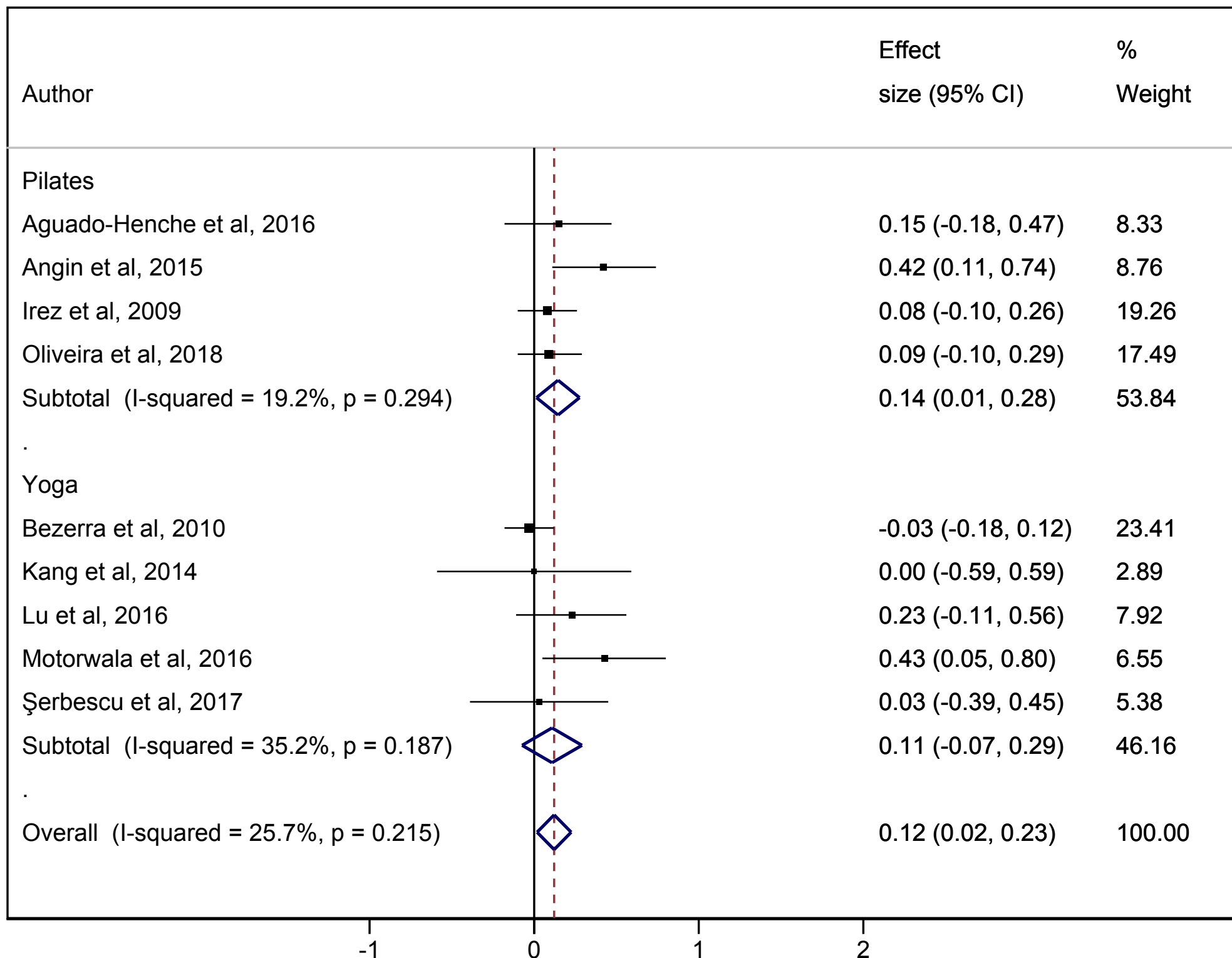

Supplement: S4 Fig — (PDF) [file pone.0251391.s004.pdf]

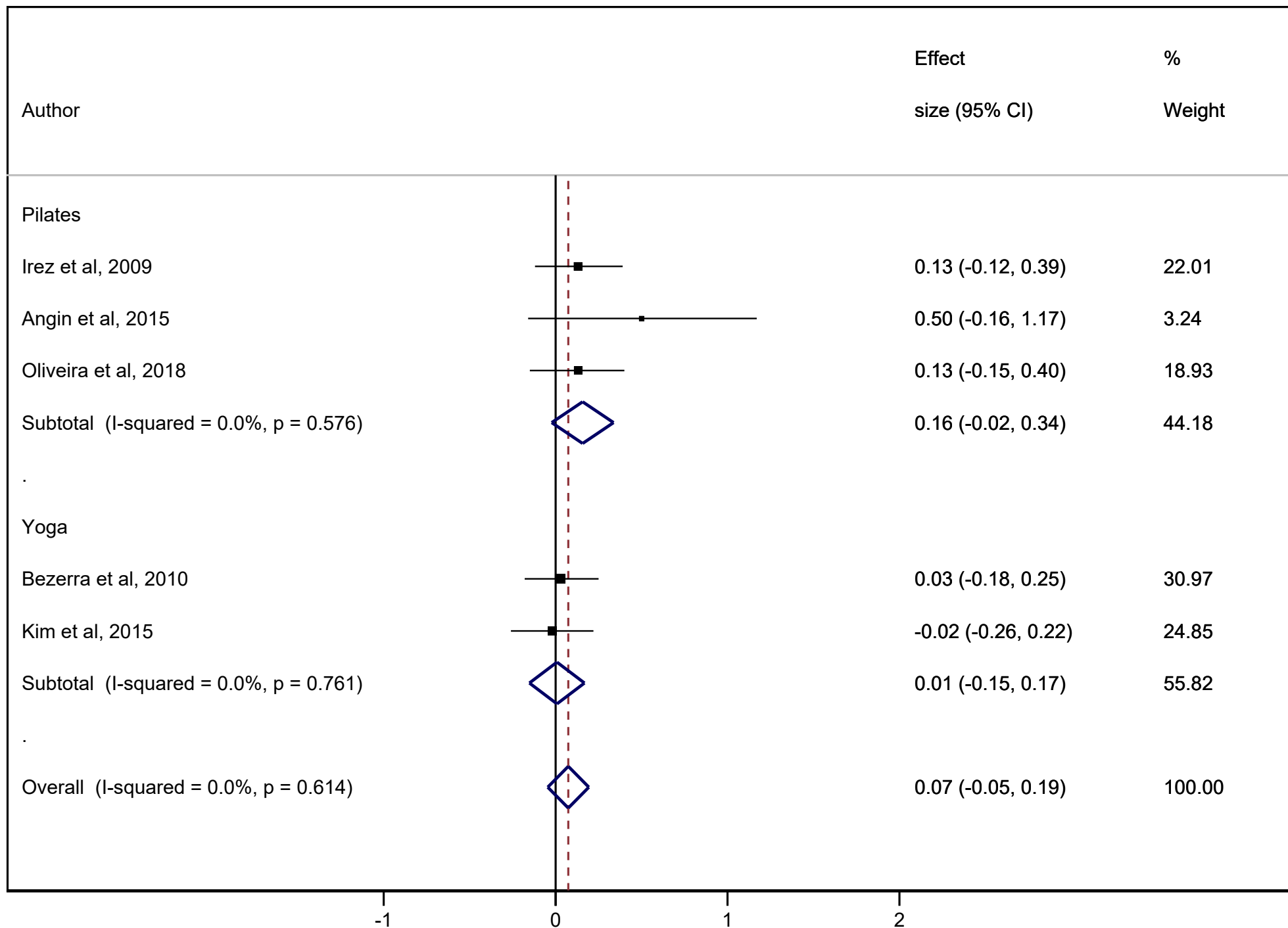

Supplement: S5 Fig — (PDF) [file pone.0251391.s005.pdf]

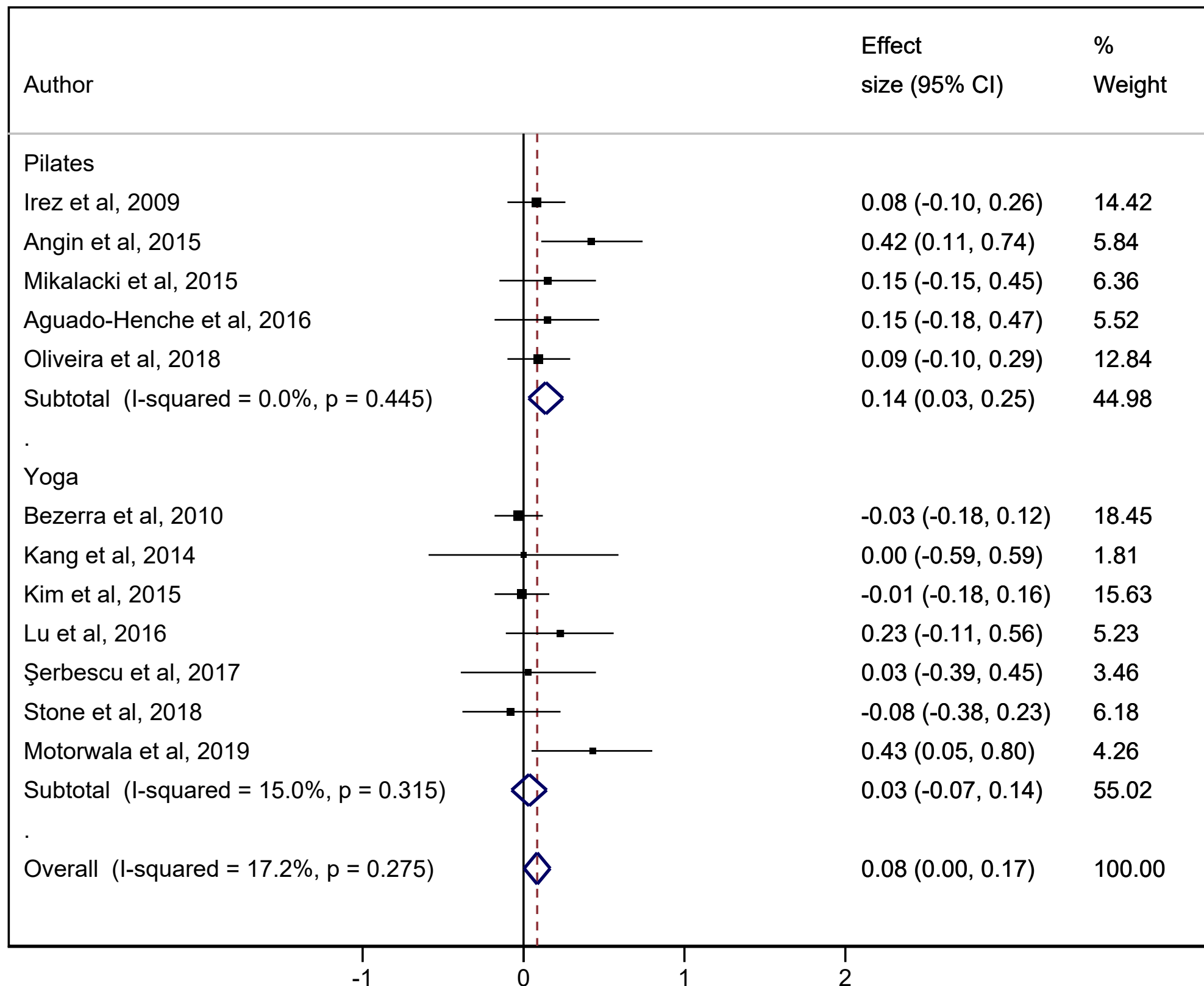

Supplement: S6 Fig — (PDF) [file pone.0251391.s006.pdf]

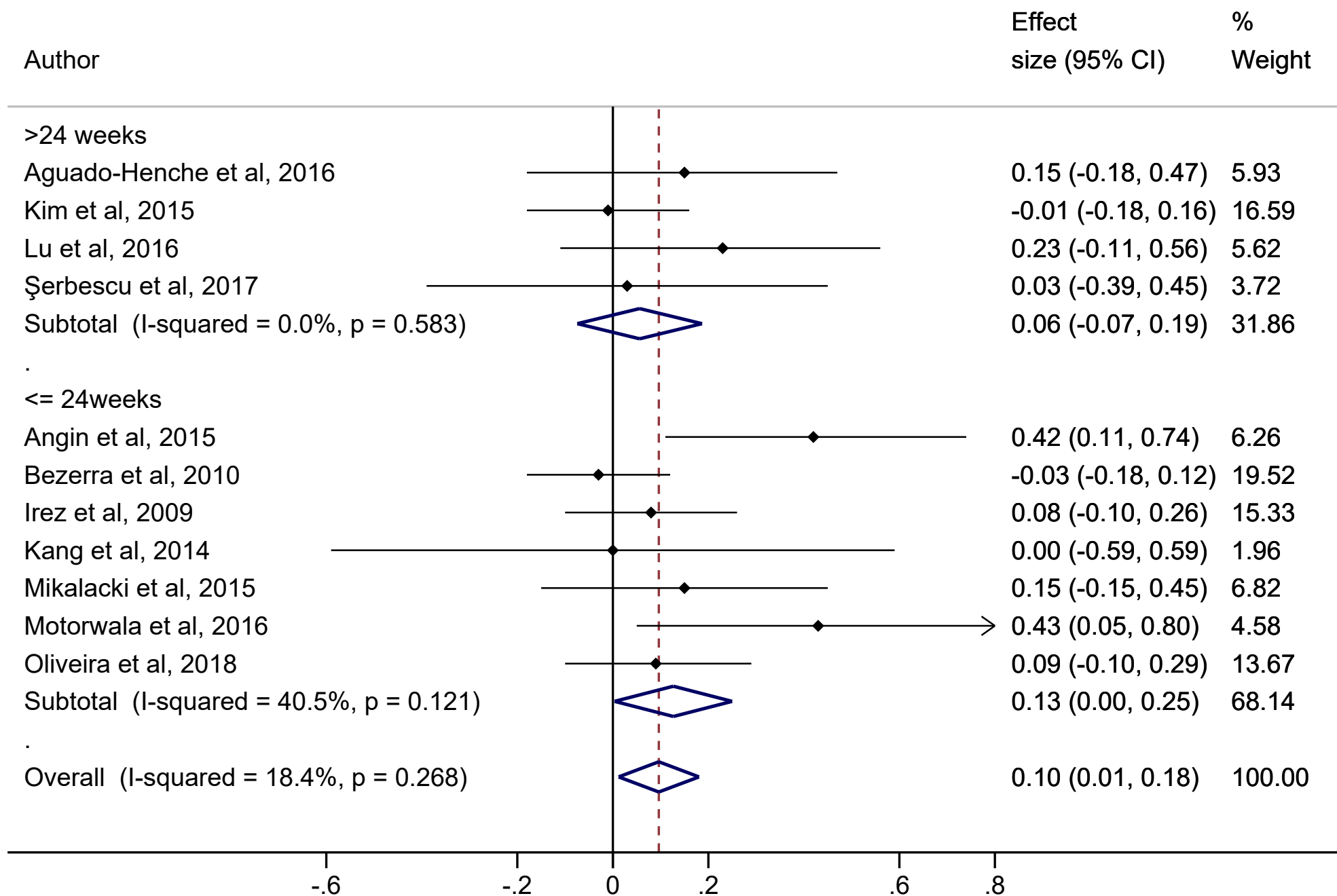

Supplement: S7 Fig — (PDF) [file pone.0251391.s007.pdf]

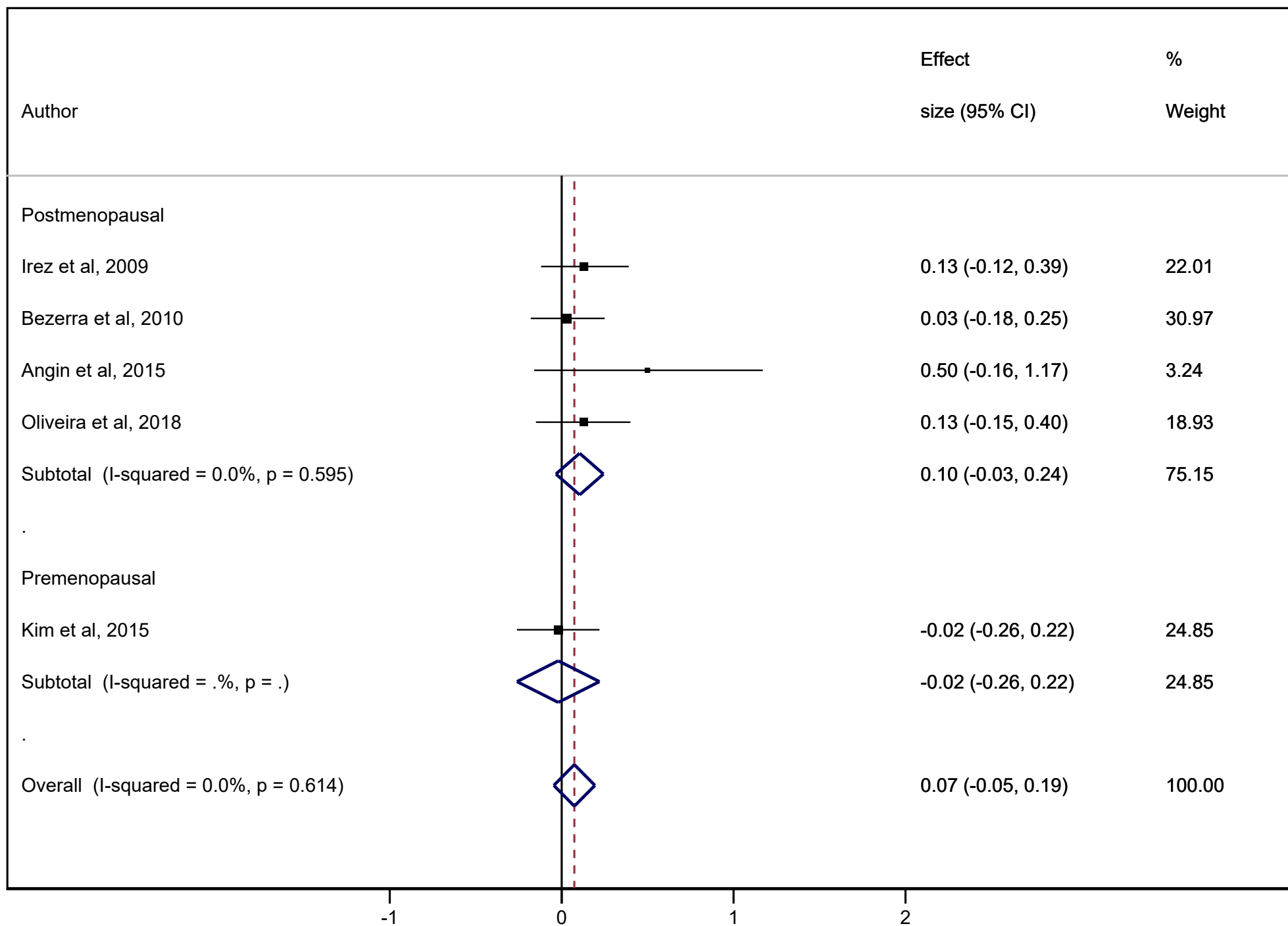

Supplement: S8 Fig — (PDF) [file pone.0251391.s008.pdf]

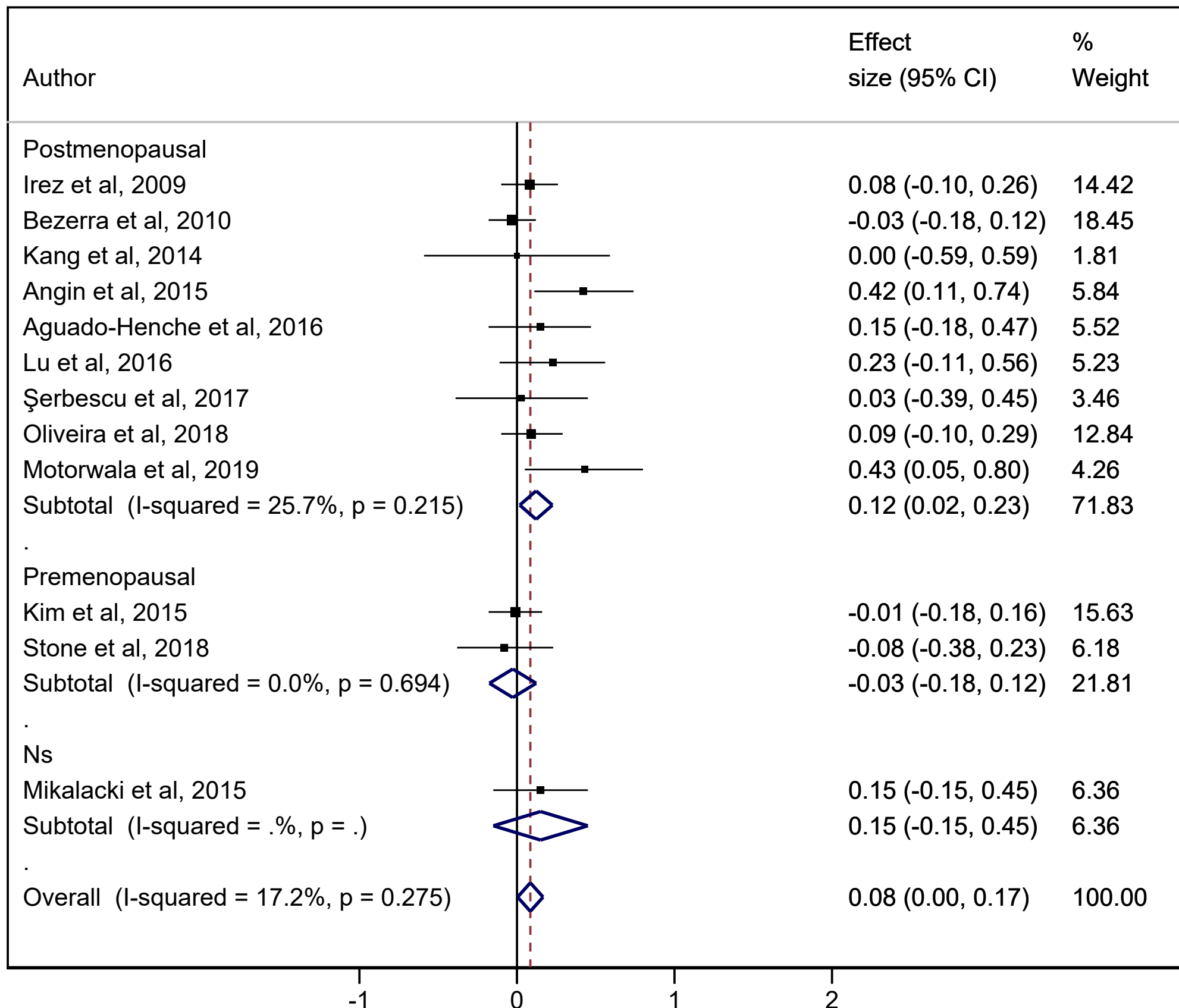

Supplement: S9 Fig — (PDF) [file pone.0251391.s009.pdf]

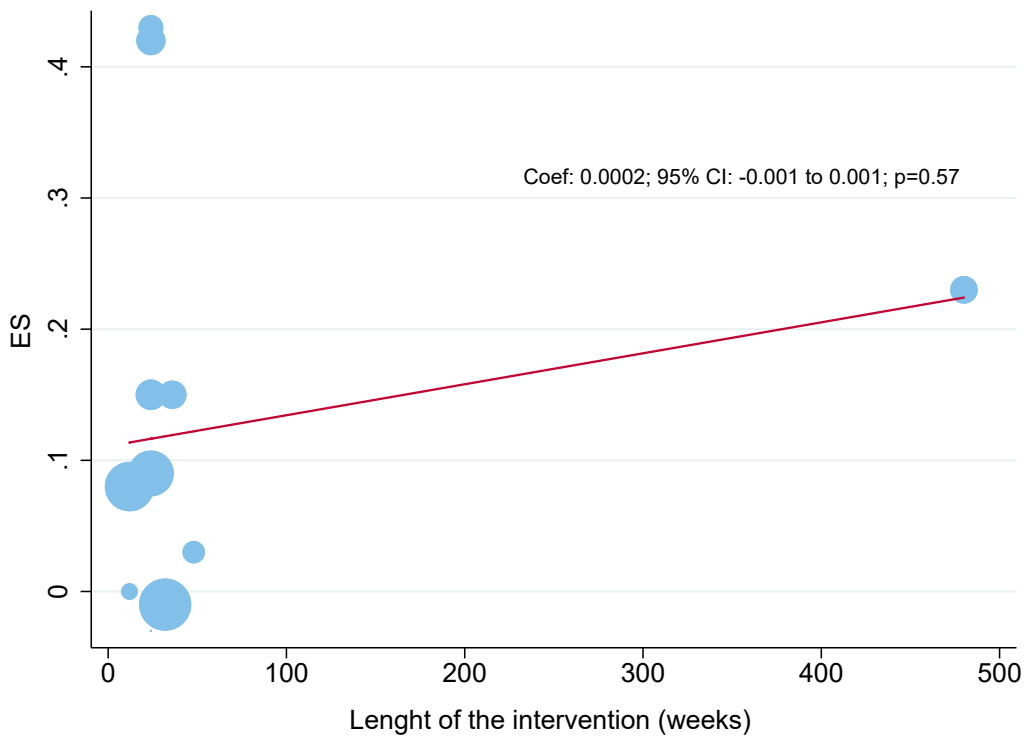

Supplement: S10 Fig — ES: Effect size; Coef: coefficient; CI: confidence interval. (PDF) [file pone.0251391.s010.pdf]
