# Supplementary material for: Effectiveness of Pilates and Yoga to improve bone density in adult women: A systematic review and meta-analysis
Source: PLoS One. 2021 May 7;16(5):e0251391. doi: 10.1371/journal.pone.0251391 (PMC8104420; doi:10.1371/journal.pone.0251391)
Supplement: S1 Table — (DOCX) [file pone.0251391.s011.docx]

**S1 Table.** Search strategy for the MEDLINE database.

| **S1 Table.** Search strategy for the MEDLINE database. | | |
| --- | --- | --- |
| **Population** | **Intervention** | **Outcome** |
| Adult  OR  Elderly  OR  Postmenopausal  OR  Premenopausal  OR  Menopausal | Pilates  OR  “Mind-body”  OR  Yoga  OR  “Exercise Movement Techniques” [Mesh] | “Bone mineral density”  OR  “Bone health”  OR  “Bone mineral mass”  OR  “T-score”  OR  DXA |
|  |  |  |
|  |  |  |
|  |  |  |
|  |  |  |
|  |  |  |
