# Supplementary material for: Effectiveness of Pilates and Yoga to improve bone density in adult women: A systematic review and meta-analysis
Source: PLoS One. 2021 May 7;16(5):e0251391. doi: 10.1371/journal.pone.0251391 (PMC8104420; doi:10.1371/journal.pone.0251391)
Supplement: S2 Table — ES: Effect Size; 95% CI: Confidence interval. (DOCX) [file pone.0251391.s012.docx]

**S2 Table**. Sensitivity analyses.

| **S2 Table**. Sensitivity analyses. | | | | |
| --- | --- | --- | --- | --- |
| **Intervention vs control group** |  |  |  |  |
| Author, year | ES | 95 % CI | | I^2^ |
| Irez et al, 2009 [34] | 0.06 | -0.08 to 0.19 | | 0 |
| Bezerra et al, 2010 [30] | 0.09 | -0.05 to 0.24 | | 0 |
| Angin et al, 2015 [17] | 0.06 | -0.06 to 0.18 | | 0 |
| Kim et al, 2015 [35] | 0.10 | -0.03 to 0.24 | | 0 |
| Oliveira et al, 2018 [18] | 0.06 | -0.07 to 0.19 | | 0 |
| **Intervention groups** |  |  |  |  |
| Author, year | ES | 95 % CI | | I^2^ |
| Irez et al, 2009 [34] | 0.11 | 0.01 to 0.21 | | 26.5 |
| Bezerra et al, 2010 [30] | 0.12 | 0.03 to 0.21 | | 4.8 |
| Kang et al, 2014 [36] | 0.10 | 0.01 to 0.19 | | 26.1 |
| Angin et al, 2015 [17] | 0.06 | -0.01 to 0.14 | | 0 |
| Kim et al, 2015 [35] | 0.12 | 0.03 to 0.21 | | 17.2 |
| Mikalacki et al, 2015 [19] | 0.10 | 0.00 to 0.19 | | 25.3 |
| Aguado-Henche et al, 2016 [33] | 0.10 | 0.01 to 0.19 | | 25.5 |
| Lu et al, 2016 [32] | 0.09 | 0.00 to 0.18 | | 21.5 |
| Motorwala et al, 2016 [31] | 0.07 | -0.00 to 0.14 | | 0 |
| Şerbescu et al, 2017 [37] | 0.10 | 0.01 to 0.19 | | 26.2 |
| Oliveira et al, 2018 [18] | 0.10 | 0.01 to 0.20 | | 26.5 |
| ES: Effect Size*;* 95 % CI: Confidence interval. | | | |  |
