# Supplementary material for: Effectiveness of Pilates and Yoga to improve bone density in adult women: A systematic review and meta-analysis
Source: PLoS One. 2021 May 7;16(5):e0251391. doi: 10.1371/journal.pone.0251391 (PMC8104420; doi:10.1371/journal.pone.0251391)
Supplement: S3 Table — aSignificant at p ≤ 0.05. (DOCX) [file pone.0251391.s013.docx]

**S3 Table**. Meta-regression analyses by age.

| **S3 Table**. Meta-regression analyses by age. | | |
| --- | --- | --- |
|  | Bias Coefficient | p |
| **Intervention vs control group** | 0.004 | 0.51 |
| **Intervention groups** | 0.002 | 0.75 |
| ^a^Significant at p ≤ 0.05. | |  |
