# Supplementary material for: Effectiveness of Pilates and Yoga to improve bone density in adult women: A systematic review and meta-analysis
Source: PLoS One. 2021 May 7;16(5):e0251391. doi: 10.1371/journal.pone.0251391 (PMC8104420; doi:10.1371/journal.pone.0251391)
Supplement: S4 Table — BMD: Bone mineral density; BMI: Body mass index. aSignificant at p ≤ 0.05. (DOCX) [file pone.0251391.s014.docx]

**S4 Table**. Meta-regression analyses by baseline BMD values after adjusting for height and for BMI.

| **S4 Table**. Meta-regression analyses by baseline BMD values after adjusting for height and for BMI. | | |
| --- | --- | --- |
|  | Bias Coefficient | p |
| **BMD/Height** |  |  |
| **Intervention vs control group** | -1.24 | 0.36 |
| **Intervention groups** | -0.71 | 0.18 |
|  |  |  |
| **BMI** |  |  |
| **Intervention vs control group** | -0.10 | 0.45 |
| **Intervention groups** | -0.02 | 0.48 |
| BMD: Bone mineral density; BMI: Body mass index.  ^a^Significant at p ≤ 0.05. | |  |
