# Supplementary material for: Effectiveness of Pilates and Yoga to improve bone density in adult women: A systematic review and meta-analysis
Source: PLoS One. 2021 May 7;16(5):e0251391. doi: 10.1371/journal.pone.0251391 (PMC8104420; doi:10.1371/journal.pone.0251391)
Supplement: S5 Table — aSignificant at p ≤ 0.1. (DOCX) [file pone.0251391.s015.docx]

**S5 Table**. Publication bias by Egger’s test.

| **S5 Table**. Publication bias by Egger’s test. | | |
| --- | --- | --- |
|  | Bias Coefficient | p |
| **Intervention vs control group** | 2.20 | 0.143 |
| **Intervention groups** | 1.38 | 0.14 |
| ^a^Significant at p < 0.1. |  |  |
