# Supplementary material for: Effectiveness of Pilates and Yoga to improve bone density in adult women: A systematic review and meta-analysis
Source: PLoS One. 2021 May 7;16(5):e0251391. doi: 10.1371/journal.pone.0251391 (PMC8104420; doi:10.1371/journal.pone.0251391)
Supplement: Attachment — Submitted filename: Response to Reviewers.docx [file pone.0251391.s017.docx]

Dear Editor-in-chief,

Thank you for reviewing our manuscript PONE-D-21-03751 entitled “Effectiveness of Pilates and Yoga to improve bone health in adult women: a systematic review and meta-analysis”.

We would like to thank you for giving us the opportunity to revise and improve our manuscript; we also thank the reviewers for their thoughtful and constructive comments.

We have considered all the suggestions and incorporated them into the revised manuscript. Changes to the original manuscript are marked in red, and we believe our manuscript is stronger as result of these modifications. An itemized point-by-point response to the reviewers’ comments is presented below.

Yours sincerely,

Dr. Celia Álvarez-Bueno.

**Academic Editor:**

Journal Requirements:

When submitting your revision, we need you to address these additional requirements.

**Academic Editor’s comment:**

1. Please ensure that your manuscript meets PLOS ONE's style requirements, including those for file naming. The PLOS ONE style templates can be found at <https://journals.plos.org/plosone/s/file?id=wjVg/PLOSOne_formatting_sample_main_body.pdf> and <https://journals.plos.org/plosone/s/file?id=ba62/PLOSOne_formatting_sample_title_authors_affiliations.pdf>

**Authors**: Thank you for the guidelines. We have formatted the manuscript to meet the PLOS ONE’s style requirements.

**Academic Editor’s comment:**

2. Please explain the reasons, and number of studies excluded for each reason, in the flow diagram. Thank you.

**Authors**: Thank you so much for the comment. We have added the number of studies and the reasons for exclusion after full-text reading in the flow diagram presented in Fig 1.

**Academic Editor’s comment:**

3. We note that the original search was performed in April 2020. Please discuss whether relevant literature has been published in the interim that would be expected to affect the results of the meta-analysis.

**Authors**: We would like to thank the academic editor’s comment. We have updated the literature search to include the most recent evidence. Unfortunately, no additional study has been found. We have properly updated the flow chart and method section.

**Academic Editor’s comment:**

4. Please include captions for your Supporting Information files at the end of your manuscript, and update any in-text citations to match accordingly. Please see our Supporting Information guidelines for more information: <http://journals.plos.org/plosone/s/supporting-information>

**Authors**: Thank you for the suggestion. We have included the required captions for Supporting Information files at the end of our manuscript, and we have also updated the in-text citations to match accordingly.

**Academic Editor’s comment:**

5. We note that this manuscript is a systematic review or meta-analysis; our author guidelines therefore require that you use PRISMA guidance to help improve reporting quality of this type of study. Please upload copies of the completed PRISMA checklist as Supporting Information with a file name “PRISMA checklist”.

**Authors**: Thank you for the recommendation. We have submitted the required PRISMA checklist file as S1_File.

**Reviewer #1:**

**Reviewer’s comment:**

1. First of all, the title of the manuscript could be change to "Effectiveness of Pilates and Yoga to improve Bone Density in adult women" instead of Bone Health which carries a broader apprehension of musculo-skeletal well-being. The only evaluation criterium used in the review is Bone Density.

**Authors**: We appreciate this reviewer’s suggestion. As suggested, we have modified the title of the manuscript:

“Effectiveness of Pilates and Yoga to improve bone mineral density in adult women: a systematic review and meta-analysis”.

**Reviewer’s comment:**

2. To increase the practical value of the review, authors should consider separately:

a) Postmenopausal reports

b) Intensity of exercises

c) Differences between Yoga and Pilates

d) Short and long durations of exercises and

e) Other physical benefits

**Authors**: We would like to thank the reviewer’s comment. We have carefully considered all the comments and incorporated them to the manuscript.

a) We have conducted an additional subgroup meta-analysis based on the menopausal status of the participants, which are available in S8 and S9 Figs. We have properly modified methods, results, and discussion sections.

“Additionally, subgroup analyses based on (…) menopausal status as well as meta-regression(...).

“Lastly, meta-analyses for interventions vs CG and for the pre-post intervention analysis according to menopausal status are available in S8 and S9 Figs.”

b) Because of the impossibility to estimate the intensity of exercises, we have included this fact as a limitation of our study.

“In this sense, it is supposed that high volume trainings lead to a smaller decrease of BMD in postmenopausal women (7) and also that the level of strain and body position during each exercise task may affect the load of the exercise impacting on BMD, (47) but these factors cannot be addressed in our study.”

c) The differences between Yoga and Pilates have been presented by subgroup analyses and discussed in discussion section.

“As afore-mentioned and in line with our data, it seems that body position and physical demands during Pilates’ exercises produced more mechanical stress on bone when compared with Yoga exercises.”

d) Aspects related with the duration of exercises have been addressed by subgroup based on the length, ≤ 24 weeks or >24 weeks, as well as meta-regression considering the weeks of the intervention. Method, result and discussion sections have been properly modified.

“Additionally, subgroup analyses based on the type of intervention (Pilates vs Yoga), length (≤ 24 weeks or >24 weeks) and menopausal status as well as meta-regression models by mean age, baseline BMD values after adjusting for height and baseline body mass index (BMI) and length were conducted to determine their potential effect on the pooled ES estimates.”

“The subgroup analysis based on the length, ≤ 24 weeks or >24 weeks, was conducted in the pre-post intervention showing a pooled ES of 0.13 (95% CI: 0.00 to 0.25; I2=40.5%) and 0.06 (95% CI: -0.07 to 0.19; I2=0.0%), respectively (S7 Fig).”

“(..) neither were the meta-regression models based on baseline BMD values after adjusting for height (p=0.36) or those based on BMI values (p=0.45) (S4 Table) or length of the intervention (p=0.57) (S10 Fig).”

“In this sense, it is well established that physical training should be maintained for at least one year to demonstrate substantial benefits in bone mass since the physiological cycle of bone remodelling lasts between four and six months,(48) and only two of the studies included in this review accomplished this.(31,36)”

e) Other physical benefits accomplished by mind-body exercises such as Pilates and Yoga have been discussed in the discussion section.

“Finally, because the type of exercise may modify the effect on BMD,(43) multicomponent strength and balance trainings have been recommended(39,44) for improving not only bone health, but also physical function in daily life activities, and for preventing falls and osteoporosis-related fractures associated with the decline of BMD”

**Reviewer’s comment:**

3. It might not be possible to include the additional data as proposed, authors could at least discuss about the variants, using information provided within or outside the systematic study.

**Authors**: Thank you for this suggestion. As it has not been possible to include some of the additional data proposed, we have discussed these items and included them as limitations of our study.

**Reviewer #2:**

In this meta-analysis, the authors investigate the effects of pilates and yoga on BMD in (mostly postmenopausal) women. They found no effect. The work is adequately performed. It was registered with PROSPERO and reported according to PRISMA guidelines. I have the following specific comments:

**Reviewer’s comment:**

1. The conclusion should not necessarily be that "additional high-quality studies with an adequate intervention length are needed to provide a more accurate picture of the evidence". I believe that yoga/Pilates is unlikely to change BMD, and the effect size here excludes a large effect. I suggest to remove this eternal "more evidence needed" mantra.

**Authors**: We appreciate this recommendation. As suggested, we have modified the sentence in the Abstract section:

“Despite of the non-significant results, the BMD maintenance in the postmenopausal population, when BMD detrimental is expected, could be understood as a positive result added to the beneficial impact of Pilates-Yoga in multiple fracture risk factors, including but not limited to, strength and balance.”

**Reviewer’s comment:**

2. Explain all abbreviations upon first use (e.g., ES, CI in the abstract)

**Authors**: We would like to apologize for this mistake. As suggested, it has been corrected.

**Reviewer’s comment:**

3. The authors should explain in the Introduction what exactly Pilates is and how it differs from Yoga.

**Authors**: We appreciate the reviewer’s suggestion. We have briefly described Pilates in the introduction section and the main differences with Yoga exercises.

“Despite of the combined classification in Mind-body techniques, Pilates and Yoga present differences that may have influence on bone. For instance, Pilates is a therapeutic exercise highly focused on core-strengthening while Yoga is more related to breathing and meditation exercises.”
